# 1,4-Dihex­yloxy-2,5-bis­(2-nitro­phen­yl)benzene

**DOI:** 10.1107/S1600536812009944

**Published:** 2012-03-10

**Authors:** Norma Wrobel, Dieter Schollmeyer, Heiner Detert

**Affiliations:** a University Mainz, Duesbergweg 10-14, 55099 Mainz, Germany

## Abstract

The title compound, C_30_H_36_N_2_O_6_, was prepared *via* twofold Suzuki coupling of a diboronic acid with bromo­nitro­benzene. The mol­ecule is located on a crystallographic inversion centre. The lateral benzene ring and the central ring make a dihedral angle of 48.75 (14)° and the nitro group is twisted by 41.47 (13)° out of the plane of the benzene ring. The nitro and hex­yloxy groups are in close proximity and the hex­yloxy chain adopts an all-*anti* conformation.

## Related literature

For the synthesis of carbazoles and heteroanalogous carbazoles, see: Letessier *et al.* (2011 ▶); Dassonneville *et al.* (2011 ▶); Nissen & Detert (2011 ▶); Letessier & Detert (2012 ▶). For the Cadogan reaction, see: Cadogan (1962 ▶). For Suzuki cross-couplings see Miyaura & Suzuki (1995 ▶). For π-systems for optoelectronic applications, see: Nemkovich *et al.* (2009 ▶). For structures of substituted *p*-terphenyls, see: Jones *et al.* (2005 ▶), Moschel *et al.* (2011 ▶). For torsion in biphenyls, see: Miao *et al.* (2009 ▶); Fischer *et al.* (2007 ▶).
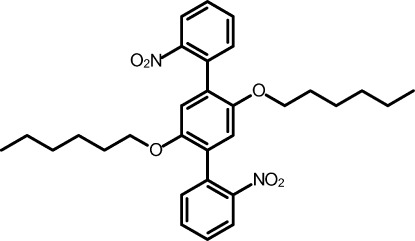


## Experimental

### 

#### Crystal data

C_30_H_36_N_2_O_6_
*M* *_r_* = 520.61
Monoclinic, 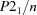

*a* = 7.9314 (4) Å
*b* = 19.2029 (17) Å
*c* = 9.1247 (5) Å
β = 96.368 (5)°
*V* = 1381.17 (16) Å^3^
*Z* = 2
Mo *K*α radiation
μ = 0.09 mm^−1^
*T* = 193 K
0.44 × 0.30 × 0.20 mm

#### Data collection

Stoe IPDS 2T diffractometer
8154 measured reflections
3331 independent reflections
2610 reflections with *I* > 2σ(*I*)
*R*_int_ = 0.026

#### Refinement

*R*[*F* ^2^ > 2σ(*F* ^2^)] = 0.044
*wR*(*F* ^2^) = 0.120
*S* = 1.07
3331 reflections
173 parameters
H-atom parameters constrained
Δρ_max_ = 0.31 e Å^−3^
Δρ_min_ = −0.27 e Å^−3^

### 

Data collection: *X-AREA* (Stoe & Cie, 2011 ▶); cell refinement: *X-AREA*; data reduction: *X-RED* (Stoe & Cie, 2011 ▶); program(s) used to solve structure: *SIR97* (Altomare *et al.*, 1999 ▶); program(s) used to refine structure: *SHELXL97* (Sheldrick, 2008 ▶); molecular graphics: *PLATON* (Spek, 2009 ▶); software used to prepare material for publication: *PLATON*.

## Supplementary Material

Crystal structure: contains datablock(s) I, global. DOI: 10.1107/S1600536812009944/bt5839sup1.cif

Structure factors: contains datablock(s) I. DOI: 10.1107/S1600536812009944/bt5839Isup2.hkl

Supplementary material file. DOI: 10.1107/S1600536812009944/bt5839Isup3.cml

Additional supplementary materials:  crystallographic information; 3D view; checkCIF report

## Figures and Tables

**Fig. 1.**
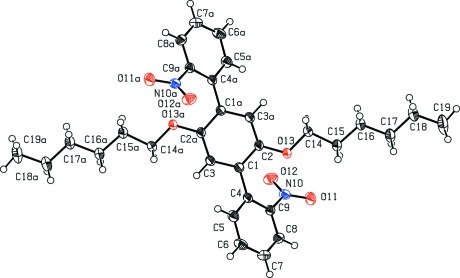
View of compound I. Displacement ellipsoids are drawn at the 50% probability level. Second part of the molecule labeled with a generated applying symmetry code 1 - x, 1 - y, 1 - z.

## supplementary crystallographic information

### Comment

As part of a larger project on the synthesis of carbazoles and heteroanalogous carbazoles (Letessier *et al.* 2011, Dassonneville *et al.* 2011, Nissen & Detert 2011, Letessier & Detert 2012) the Cadogan reaction (Cadogan 1962) appeared to be a suitable method for the construction of larger planar π-systems for optoelectronic applications (Nemkovich *et al.* 2009). The title compond was prepared as an intermediate for the synthesis of dihexyloxy-indolocarbazole.

The title compound crystallizes in a centrosymmetrical conformation with a highly twisted dinitroterphenyl core and hexyloxy chains in an *all-anti* conformation. The dihedral angle of the mean planes of the central and the lateral ring is 131.25 (14)° with the *ortho*-substituents nitro- and hexyloxy in close proximity. The distance N10 - O13 (nitro-hexyloxy) is only 2.710 (2) Å. The nitro group is twisted out of the plane of the adjacent benzene ring, the dihedral angle is 138.53 (13)° pointing towards the adjacent *o*-hexyloxy group. A *o*-methyl substitution on on a biphenyl linkage is sufficient to open the dihedral angle from 9.45 ° (Fischer *et al.* 2007) to more than 63° (Jones *et al.* 2005). The twist (131.25°) found in the title compound - though *o*,*o*-disubstituted on both biphenyl linkages - is significantly smaller. This can result from an electronic attraction between N10 (nitro) and O13 (hexyloxy). Miao *et al.* (2009) reported a dihedral angle of 60.5° in the fourfold *o*-substituted 2,2-dimethoxy-6,6-dinitrobiphenyl.

### Experimental

Synthesis: A mixture of 2,5-dihexyloxy-1,4-phenylenediboronic acid (500 mg, 1.37 mmol), 1-bromo-2-nitrobenzene (553 mg, 2.74 mmol), Pd(PPh_3_)_3_ (79 mg, 0.067 mmol) in dimethoxyethane (10 ml) was stirred for 45 min at 298 K. An aqueous solution of Na_2_CO_3_ (1*M*, 8.2 ml) was added and the mixture heated to 353 K for 18 h. The cooled mixture was poured into water (40 ml) and the product was isolated by extraction with dichloromethane (3 *x* 15 ml), washing the pooled solutions with brine (2 *x* 10 ml), drying (Na_2_SO_4_) and crystallization from chloroform/pentane. Yield: 495 mg (70%) of a yellow solid with m. p. 438 - 440 K. *R*_f_ = 0.41 (silica gel, petroleum ether/ethyl acetate 9/1).

### Refinement

Hydrogen atoms were placed at calculated positions with C—H = 0.95 Å (aromatic) or 0.98–0.99 Å (*sp*^3^ C-atom). All H atoms were refined in the riding-model approximation with isotropic displacement parameters set at 1.2–1.5 times of the *U*_eq_ of the parent atom.

### Crystal data

**Table:**

C_30_H_36_N_2_O_6_	*F*(000) = 556
*M**_r_* = 520.61	*D*_x_ = 1.252 Mg m^−^^3^
Monoclinic, *P*2_1_/*n*	Mo *K*α radiation, λ = 0.71073 Å
Hall symbol: -P 2yn	Cell parameters from 7928 reflections
*a* = 7.9314 (4) Å	θ = 3.2–29.1°
*b* = 19.2029 (17) Å	µ = 0.09 mm^−^^1^
*c* = 9.1247 (5) Å	*T* = 193 K
β = 96.368 (5)°	Block, yellow
*V* = 1381.17 (16) Å^3^	0.44 × 0.30 × 0.20 mm
*Z* = 2	

### Data collection

**Table:**

Stoe IPDS 2T diffractometer	2610 reflections with *I* > 2σ(*I*)
Radiation source: sealed X-ray tube, 12 x 0.4 mm long-fine focus	*R*_int_ = 0.026
Plane graphite monochromator	θ_max_ = 28.0°, θ_min_ = 3.2°
Detector resolution: 6.67 pixels mm^-1^	*h* = −10→10
ω scan	*k* = −25→22
8154 measured reflections	*l* = −12→10
3331 independent reflections	

### Refinement

**Table:**

Refinement on *F*^2^	Primary atom site location: structure-invariant direct methods
Least-squares matrix: full	Secondary atom site location: difference Fourier map
*R*[*F*^2^ > 2σ(*F*^2^)] = 0.044	Hydrogen site location: inferred from neighbouring sites
*wR*(*F*^2^) = 0.120	H-atom parameters constrained
*S* = 1.07	*w* = 1/[σ^2^(*F*_o_^2^) + (0.0562*P*)^2^ + 0.4038*P*] where *P* = (*F*_o_^2^ + 2*F*_c_^2^)/3
3331 reflections	(Δ/σ)_max_ < 0.001
173 parameters	Δρ_max_ = 0.31 e Å^−^^3^
0 restraints	Δρ_min_ = −0.27 e Å^−^^3^

### Special details

**Table:**

Experimental. ^1^H-NMR (400 MHz, CDCl_3_): δ = 7.95 (dd, ^3^J = 8.5 Hz, ^4^J= 1.2 Hz, 2 H, 3-H); 7.64 (dt, ^3^J = 7.5 Hz, ^4^J = 1.4 Hz, 2 H, 4-H); 7.49 - 7.45 (m, 4 H); 6.83 (s, 2 H, 2-H); 3.81 (bs (*t*), 4 H, O—CH_2_); 1.59 - 1.54 (m, 4 H); 1.25 - 1.18 (m, 12 H); 0.80 (t, ^3^J = 6.9 Hz, 6 H, CH_3_).^13^C-NMR (75 MHz, CDCl_3_): δ = 149.7 (s, 2-C), 149.5 (*s*), 132.9 (*s*), 132.6 (*d*), 132.5 (*d*), 128.1 (*d*), 127.8 (*s*), 123.9 (*d*), 113.4 (*d*), 69.1 (*t*), 31.3 (*t*), 28.8 (*t*), 25.4 (*t*), 22.5 (*t*), 13.8 (*q*).IR (ATR): ν = 3734, 3585, 3070, 2944, 2869, 2855, 2363, 2334, 1608, 1573, 1530, 1510, 1469, 1441, 1387, 1358, 1290, 1255, 1209, 1165, 1144, 1025, 997, 870, 860.MS (EI): m/*z* = 520 (100%, *M*^+.^).
Geometry. All e.s.d.'s (except the e.s.d. in the dihedral angle between two l.s. planes) are estimated using the full covariance matrix. The cell e.s.d.'s are taken into account individually in the estimation of e.s.d.'s in distances, angles and torsion angles; correlations between e.s.d.'s in cell parameters are only used when they are defined by crystal symmetry. An approximate (isotropic) treatment of cell e.s.d.'s is used for estimating e.s.d.'s involving l.s. planes.
Refinement. Refinement of *F*^2^ against ALL reflections. The weighted *R*-factor *wR* and goodness of fit *S* are based on *F*^2^, conventional *R*-factors *R* are based on *F*, with *F* set to zero for negative *F*^2^. The threshold expression of *F*^2^ > σ(*F*^2^) is used only for calculating *R*-factors(gt) *etc*. and is not relevant to the choice of reflections for refinement. *R*-factors based on *F*^2^ are statistically about twice as large as those based on *F*, and *R*- factors based on ALL data will be even larger.

### Fractional atomic coordinates and isotropic or equivalent isotropic displacement parameters (Å^2^)

**Table:**

	*x*	*y*	*z*	*U*_iso_*/*U*_eq_	
C1	0.46215 (15)	0.52525 (7)	0.35512 (14)	0.0240 (3)	
C2	0.35820 (15)	0.54228 (7)	0.46442 (14)	0.0252 (3)	
C3	0.60362 (16)	0.48321 (8)	0.39358 (14)	0.0264 (3)	
H3	0.6756	0.4717	0.3206	0.032*	
C4	0.43305 (15)	0.55452 (7)	0.20336 (14)	0.0232 (3)	
C5	0.57076 (16)	0.58310 (8)	0.14150 (15)	0.0299 (3)	
H5	0.6801	0.5811	0.1956	0.036*	
C6	0.55321 (18)	0.61424 (8)	0.00394 (16)	0.0338 (3)	
H6	0.6502	0.6318	−0.0362	0.041*	
C7	0.39481 (18)	0.61991 (8)	−0.07522 (16)	0.0309 (3)	
H7	0.3820	0.6432	−0.1676	0.037*	
C8	0.25493 (17)	0.59149 (7)	−0.01925 (15)	0.0273 (3)	
H8	0.1456	0.5945	−0.0732	0.033*	
C9	0.27660 (15)	0.55862 (7)	0.11640 (14)	0.0225 (3)	
N10	0.12608 (13)	0.52343 (6)	0.16019 (12)	0.0271 (3)	
O11	−0.01207 (12)	0.55180 (6)	0.13048 (12)	0.0381 (3)	
O12	0.14470 (14)	0.46637 (6)	0.21901 (12)	0.0380 (3)	
O13	0.22425 (12)	0.58564 (6)	0.42244 (10)	0.0314 (2)	
C14	0.11269 (18)	0.60345 (9)	0.52871 (16)	0.0330 (3)	
H14A	0.1748	0.6300	0.6106	0.040*	
H14B	0.0665	0.5607	0.5699	0.040*	
C15	−0.02962 (17)	0.64694 (9)	0.45359 (16)	0.0330 (3)	
H15A	0.0189	0.6874	0.4055	0.040*	
H15B	−0.0946	0.6188	0.3758	0.040*	
C16	−0.1481 (2)	0.67270 (12)	0.5597 (2)	0.0560 (6)	
H16A	−0.1793	0.6328	0.6201	0.067*	
H16B	−0.0862	0.7067	0.6274	0.067*	
C17	−0.30865 (18)	0.70652 (8)	0.49024 (18)	0.0344 (3)	
H17A	−0.2781	0.7465	0.4299	0.041*	
H17B	−0.3715	0.6726	0.4231	0.041*	
C18	−0.4239 (3)	0.73189 (14)	0.5993 (3)	0.0670 (7)	
H18A	−0.4492	0.6924	0.6631	0.080*	
H18B	−0.3626	0.7675	0.6632	0.080*	
C19	−0.5889 (2)	0.76264 (11)	0.5319 (3)	0.0602 (6)	
H19A	−0.5662	0.8048	0.4767	0.090*	
H19B	−0.6592	0.7745	0.6101	0.090*	
H19C	−0.6488	0.7287	0.4650	0.090*	

### Atomic displacement parameters (Å^2^)

**Table:**

	*U*^11^	*U*^22^	*U*^33^	*U*^12^	*U*^13^	*U*^23^
C1	0.0190 (5)	0.0341 (7)	0.0187 (6)	0.0020 (5)	0.0005 (4)	−0.0030 (5)
C2	0.0172 (5)	0.0358 (7)	0.0218 (6)	0.0056 (5)	−0.0005 (4)	−0.0031 (5)
C3	0.0204 (6)	0.0391 (7)	0.0199 (6)	0.0049 (5)	0.0027 (5)	−0.0037 (5)
C4	0.0202 (5)	0.0296 (6)	0.0198 (6)	0.0027 (5)	0.0023 (4)	−0.0032 (5)
C5	0.0189 (6)	0.0436 (8)	0.0271 (7)	−0.0031 (5)	0.0017 (5)	−0.0039 (6)
C6	0.0302 (7)	0.0441 (8)	0.0285 (7)	−0.0111 (6)	0.0094 (6)	−0.0021 (6)
C7	0.0375 (7)	0.0326 (7)	0.0229 (6)	−0.0045 (6)	0.0040 (5)	0.0024 (5)
C8	0.0260 (6)	0.0322 (7)	0.0228 (6)	0.0003 (5)	−0.0010 (5)	0.0010 (5)
C9	0.0186 (5)	0.0268 (6)	0.0219 (6)	−0.0002 (5)	0.0020 (4)	−0.0012 (5)
N10	0.0210 (5)	0.0398 (7)	0.0201 (5)	−0.0036 (5)	0.0009 (4)	−0.0006 (5)
O11	0.0178 (4)	0.0605 (7)	0.0353 (6)	0.0027 (4)	0.0006 (4)	−0.0021 (5)
O12	0.0358 (5)	0.0425 (6)	0.0351 (6)	−0.0098 (5)	0.0014 (4)	0.0114 (5)
O13	0.0239 (4)	0.0492 (6)	0.0213 (5)	0.0155 (4)	0.0029 (4)	0.0003 (4)
C14	0.0277 (6)	0.0471 (9)	0.0254 (7)	0.0141 (6)	0.0078 (5)	0.0025 (6)
C15	0.0248 (6)	0.0453 (8)	0.0288 (7)	0.0118 (6)	0.0030 (5)	0.0009 (6)
C16	0.0502 (10)	0.0811 (14)	0.0402 (9)	0.0427 (10)	0.0203 (8)	0.0197 (9)
C17	0.0254 (6)	0.0330 (7)	0.0459 (9)	0.0059 (6)	0.0090 (6)	0.0019 (6)
C18	0.0543 (11)	0.0902 (16)	0.0615 (13)	0.0435 (11)	0.0297 (10)	0.0211 (12)
C19	0.0324 (8)	0.0556 (12)	0.0951 (17)	0.0150 (8)	0.0188 (10)	0.0009 (11)

### Geometric parameters (Å, º)

**Table:**

C1—C3	1.3952 (18)	O13—C14	1.4250 (15)
C1—C2	1.4014 (17)	C14—C15	1.5065 (19)
C1—C4	1.4886 (18)	C14—H14A	0.9900
C2—O13	1.3705 (15)	C14—H14B	0.9900
C2—C3^i^	1.3867 (19)	C15—C16	1.506 (2)
C3—C2^i^	1.3867 (19)	C15—H15A	0.9900
C3—H3	0.9500	C15—H15B	0.9900
C4—C5	1.3963 (18)	C16—C17	1.505 (2)
C4—C9	1.3990 (17)	C16—H16A	0.9900
C5—C6	1.383 (2)	C16—H16B	0.9900
C5—H5	0.9500	C17—C18	1.505 (2)
C6—C7	1.382 (2)	C17—H17A	0.9900
C6—H6	0.9500	C17—H17B	0.9900
C7—C8	1.3838 (19)	C18—C19	1.503 (3)
C7—H7	0.9500	C18—H18A	0.9900
C8—C9	1.3831 (18)	C18—H18B	0.9900
C8—H8	0.9500	C19—H19A	0.9800
C9—N10	1.4655 (16)	C19—H19B	0.9800
N10—O12	1.2218 (16)	C19—H19C	0.9800
N10—O11	1.2268 (15)		
			
C3—C1—C2	118.44 (12)	O13—C14—H14B	110.0
C3—C1—C4	119.33 (11)	C15—C14—H14B	110.0
C2—C1—C4	122.08 (11)	H14A—C14—H14B	108.4
O13—C2—C3^i^	123.90 (11)	C16—C15—C14	112.27 (12)
O13—C2—C1	116.22 (11)	C16—C15—H15A	109.2
C3^i^—C2—C1	119.85 (12)	C14—C15—H15A	109.2
C2^i^—C3—C1	121.70 (11)	C16—C15—H15B	109.2
C2^i^—C3—H3	119.2	C14—C15—H15B	109.2
C1—C3—H3	119.2	H15A—C15—H15B	107.9
C5—C4—C9	115.65 (12)	C17—C16—C15	115.45 (14)
C5—C4—C1	118.49 (11)	C17—C16—H16A	108.4
C9—C4—C1	125.82 (11)	C15—C16—H16A	108.4
C6—C5—C4	122.16 (12)	C17—C16—H16B	108.4
C6—C5—H5	118.9	C15—C16—H16B	108.4
C4—C5—H5	118.9	H16A—C16—H16B	107.5
C7—C6—C5	120.15 (12)	C18—C17—C16	114.11 (15)
C7—C6—H6	119.9	C18—C17—H17A	108.7
C5—C6—H6	119.9	C16—C17—H17A	108.7
C6—C7—C8	119.71 (13)	C18—C17—H17B	108.7
C6—C7—H7	120.1	C16—C17—H17B	108.7
C8—C7—H7	120.1	H17A—C17—H17B	107.6
C9—C8—C7	119.02 (12)	C19—C18—C17	114.93 (19)
C9—C8—H8	120.5	C19—C18—H18A	108.5
C7—C8—H8	120.5	C17—C18—H18A	108.5
C8—C9—C4	123.21 (12)	C19—C18—H18B	108.5
C8—C9—N10	115.49 (11)	C17—C18—H18B	108.5
C4—C9—N10	121.17 (11)	H18A—C18—H18B	107.5
O12—N10—O11	123.84 (12)	C18—C19—H19A	109.5
O12—N10—C9	118.09 (11)	C18—C19—H19B	109.5
O11—N10—C9	118.01 (12)	H19A—C19—H19B	109.5
C2—O13—C14	118.44 (10)	C18—C19—H19C	109.5
O13—C14—C15	108.30 (11)	H19A—C19—H19C	109.5
O13—C14—H14A	110.0	H19B—C19—H19C	109.5
C15—C14—H14A	110.0		
			
C3—C1—C2—O13	177.82 (12)	C7—C8—C9—N10	−173.60 (12)
C4—C1—C2—O13	2.17 (19)	C5—C4—C9—C8	−3.0 (2)
C3—C1—C2—C3^i^	−0.7 (2)	C1—C4—C9—C8	174.57 (13)
C4—C1—C2—C3^i^	−176.30 (13)	C5—C4—C9—N10	172.61 (12)
C2—C1—C3—C2^i^	0.7 (2)	C1—C4—C9—N10	−9.8 (2)
C4—C1—C3—C2^i^	176.44 (13)	C8—C9—N10—O12	138.53 (13)
C3—C1—C4—C5	−44.37 (18)	C4—C9—N10—O12	−37.39 (18)
C2—C1—C4—C5	131.25 (14)	C8—C9—N10—O11	−38.71 (17)
C3—C1—C4—C9	138.15 (14)	C4—C9—N10—O11	145.37 (13)
C2—C1—C4—C9	−46.2 (2)	C3^i^—C2—O13—C14	−2.8 (2)
C9—C4—C5—C6	0.8 (2)	C1—C2—O13—C14	178.78 (13)
C1—C4—C5—C6	−176.95 (13)	C2—O13—C14—C15	−176.02 (12)
C4—C5—C6—C7	2.1 (2)	O13—C14—C15—C16	−175.95 (16)
C5—C6—C7—C8	−2.9 (2)	C14—C15—C16—C17	−170.43 (16)
C6—C7—C8—C9	0.8 (2)	C15—C16—C17—C18	−179.81 (19)
C7—C8—C9—C4	2.2 (2)	C16—C17—C18—C19	−177.1 (2)

Symmetry code: (i) −*x*+1, −*y*+1, −*z*+1.
